# Necroptosis Interfaces with MOMP and the MPTP in Mediating Cell Death

**DOI:** 10.1371/journal.pone.0130520

**Published:** 2015-06-10

**Authors:** Jason Karch, Onur Kanisicak, Matthew J. Brody, Michelle A. Sargent, Demetria M. Michael, Jeffery D. Molkentin

**Affiliations:** 1 Department of Pediatrics, Cincinnati Children’s Hospital Medical Center, University of Cincinnati, Cincinnati, Ohio, United States of America; 2 Howard Hughes Medical Institute, Cincinnati, Ohio, United States of America; Roswell Park Cancer Institute, UNITED STATES

## Abstract

During apoptosis the pro-death Bcl-2 family members Bax and Bak induce mitochondrial outer membrane permeabilization (MOMP) to mediate cell death. Recently, it was shown that Bax and Bak are also required for mitochondrial permeability transition pore (MPTP)-dependent necrosis, where, in their non-oligomeric state, they enhance permeability characteristics of the outer mitochondrial membrane. Necroptosis is another form of regulated necrosis involving the death receptors and receptor interacting protein kinases (RIP proteins, by *Ripk* genes). Here, we show cells or mice deficient for Bax/Bak or cyclophilin D, a protein that regulates MPTP opening, are resistant to cell death induced by necroptotic mediators. We show that Bax/Bak oligomerization is required for necroptotic cell death and that this oligomerization reinforces MPTP opening. Mechanistically, we observe mixed lineage kinase domain-like (MLKL) protein and cofilin-1 translocation to the mitochondria following necroptosis induction, while expression of the mitochondrial matrix isoform of the antiapoptotic Bcl-2 family member, myeloid cell leukemia 1 (Mcl-1), is significantly reduced. Some of these effects are lost with necroptosis inhibition in *Bax/Bak1* double null, *Ppif^-/-^*, or *Ripk3^-/-^* fibroblasts. Hence, downstream mechanisms of cell death induced by necroptotic stimuli utilize both Bax/Bak to generate apoptotic pores in the outer mitochondrial membrane as well as MPTP opening in association with known mitochondrial death modifying proteins.

## Introduction

During apoptosis the Bcl-2 family members Bax and Bak form hetero- and homo- oligomers within the mitochondrial outer membrane leading to release of cytochrome *c* and other apoptosis inducing proteins [1]. During regulated necrosis the mitochondrial permeability transition pore (MPTP) opens due to increased reactive oxygen species (ROS) and calcium in the matrix of the mitochondria. The MPTP is an inner mitochondrial membrane event that when opened leads to mitochondrial depolarization and if sustained, organelle rupture [2]. Although the actual pore forming component of the MPTP remains an area of ongoing investigation, cyclophilin D (CypD) is a known regulator of MPTP opening and necrotic cell death [2]. Indeed, *Ppif* ^*-/-*^ (CypD encoding gene) cells are resistant to necrotic stressors such as calcium overload induced by ionomycin, ROS induced by H_2_O_2_, or in vivo in response to ischemic injury [3, 4]. However, loss of the *Ppif* gene does not protect from apoptotic-induced cell death, such as with staurosporine stimulation [3, 4].

*Bax* and *Bak1* (encodes Bak protein) gene deficient cells are resistant to both apoptosis and regulated necrosis [5–7]. While Bax and Bak are responsible for apoptotic cell death by inducing mitochondrial outer membrane permeability (MOMP) and cytochrome *c* release, recently it was shown that they are also required for MPTP-dependent necrotic cell death by generating a level of permeability in the outer mitochondrial membrane in their non-oligomerized forms [6]. Bax/Bak were also recently implicated as necessary mediators of another type of cell death known as necroptosis, although the mechanism underlying this observation was not described [8, 9], which we have investigated here. Necroptosis is a specific form of regulated necrosis that occurs in response to caspase inhibition and simultaneous death receptor stimulation, such as through the tumor necrosis factor-α (TNFα) receptor [10]. Upon TNFα receptor stimulation caspase 8 becomes activated causing inhibition of receptor interacting protein kinase 1 (RIP1) [11]. If caspase 8 is inhibited during death receptor stimulation, RIP1 is activated where it binds to RIP3 and causes its activation and subsequent necroptotic cell death [12, 13]. One known substrate of the RIPs that is required for necroptosis is mix lineage kinase domain-like (MLKL), as *Mlkl* null cells are resistant to necroptotic cell death [14, 15], although how this mechanistically leads to cell death is not understood. Morphologically, necroptosis shares similar features with MPTP-dependent necrosis, such as mitochondrial swelling and early plasma membrane rupture [10].

The involvement of mitochondria, Bax/Bak or the MPTP as downstream mediators of necroptosis is a point of contention in the literature [16]. Some studies have implicated Bax/Bak and the MPTP in playing an important role in necroptosis [8, 9, 17], while other studies have suggested no involvement [18, 19]. Here, we show that Bax/Bak are required mediators of necroptosis through a mechanism involving MOMP, which then secondarily enhances MPTP-dependent cellular necrosis. Furthermore, we show that MLKL and cofilin-1 translocate to the mitochondria following a necroptotic stimuli. Cofilin-1 translocation to the mitochondria is known to induce MPTP opening and cell death [20–22]. Necroptosis also causes a decrease in the mitochondrial matrix isoform of myeloid cell leukemia 1 (Mcl-1), an anti-apoptotic Bcl-2 family member that leads to mitochondrial dysfunction when deleted [23]. A model is proposed whereby the upstream regulators of necroptosis feed into known regulators of mitochondrial dependent cell death, such as Bax/Bak and the MPTP, thus suggesting that mitochondrial dysfunction is required for effective necroptosis.

## Materials and Methods

### Animal models

*Ppif* ^*-/-*^ mice were described previously [3]. For the pancreatitis model, male mice 6–8 weeks old received intraperitoneal injections of 50 μg/kg caerulein (Sigma) or vehicle control every hour for 6 hours and sacrificed by CO_2_ inhalation 2 hours following the last injection (n = 5 each group). The pancreases were fixed in 4% paraformaldehyde, sectioned, and stained with H&E to determine damaged areas by microscopy.

### Ethics statement

All animal experimentation was approved by the Office of Research Compliance and Regulatory Affairs and by the Cincinnati Children’s Hospital Institutional Animal Care and Use Committee (Protocol Number: 2E11104). No surgical procedures were used nor were other procedures used that caused suffering. No human subjects were used.

### Tissue culture and analysis of cell death and viability

All cells were cultured in IMDM medium supplemented with 10% bovine growth serum, antibiotics, and nonessential amino acids. DKO MEFs stably expressing WT Bax or Bak or mutant Bax were described previously [24, 25]. Clonal cell lines were created by plating 0.5 cells/well onto a 96-well plate. After 10 days cells were expanded and western blots were performed to determine the expression level of Bax or Bak. *Ppif* null MEFs were described previously [6]. *Rip3k* null MEFs were described previously [26]. At 80% confluence, MEFs were treated with 200 nM staurosporine for 24 hr, 20 μM ionomycin for 24 hr, or 10 ng/mL TNFα and 50 μM caspase inhibitor zVAD-fmk for 24 hr. In some experiments, cells were pretreated with 20 μM ABT-737, or 10 μM Gossypol. Cell death was determined by the Muse Count & Viability assay (Millipore). Briefly, cells were trypsinized and washed twice and incubated with Muse Count & Viability reagent. The cells were then quantified on a Muse cell analyzer (Millipore) at 5,000 counts per sample.

### Mitochondrial isolation and analyses

Liver mitochondria were isolated by homogenization with a Teflon homogenizer followed by differential centrifugation. Mitochondrial calcium uptake was measured with Calcium Green-5N (Invitrogen) as previously described [6]. Briefly, mitochondria were isolated in MS-EGTA buffer (225 mM mannitol, 75 mM sucrose, 5 mM HEPES, and 1 mM EGTA, pH 7.4). Mitochondria (200 μg) were pretreated with 1 μg tBID or vehicle (boiled tBID) for 15 minutes at 37°C and then were incubated in KCl buffer (125 mM KCl, 20 mM HEPES, 2 mM MgCl_2_, 2 mM KH_2_PO_4_, and 40 μM EGTA, pH 7.2) containing 200 nM Calcium Green-5N, 7 mM pyruvate, and 1 mM malate. After a baseline read was recorded, mitochondria were treated with 1 pulse of 250 μM CaCl_2_. Fluorescence was quantified using a Synergy 2 Multi-Mode Microplate Reader (BioTek). Mitochondria from MEFs were isolated by homogenization with a glass homogenizer followed by differential centrifugation. For some experiments the cytoplasmic fraction was preserved and subjected to 3 additional high speed (>14000 g) centrifugations to further purify the cytoplasmic proteins.

### Electron and fluorescent microscopy

Electron microscopy was performed on WT, DKO, and *Ppif* null MEFs. Prior to fixation, cells were treated for 2 or 12 hrs with TNFα and zVAD-fmk or vehicle. Samples were then fixed in glutaraldehyde and cacodylate, embedded in epoxy resin, sectioned, and counterstained with uranyl acetate and lead citrate. Fluorescent microscopy was performed on WT MEFs. Prior to fixation, cells were treated with TNFα and zVAD-fmk or vehicle for 3 hours. Samples were then fixed in 4% paraformaldehyde and subjected to immunocytochemistry for MLKL (EMD Millipore) and SAMM50 as a mitochondrial marker (Sigma-Aldrich).

### Western blotting

MEFs or isolated mitochondria were homogenized in RIPA buffer containing protease inhibitor cocktails (Roche). After protein concentrations were measured SDS sample buffer was added and western blots were performed. For the Bak oligomerization assay WT and DKO MEFs were treated with TNFα and zVAD-FMK for 6 hours. Mitochondria were isolated as described above and suspended in tris-buffered saline containing 1% DDM and 1% digitonin. After the protein concentrations were assessed NativePAGE sample buffer (Life Technologies) was added and a western blot was performed. The following antibodies were used: Bax (Santa Cruz), Bak (Millipore), CypD (Abcam), RIP1 (Santa Cruz), RIP3 (Abcam), gapdh (Fitzgerald), BID (R&D Systems), Bcl-2 (Santa Cruz), Mcl-1(Rockland), MLKL (EMD Millipore), cofilin-1 (Santa Cruz), complex V/III (MitoProfile Total OXPHOS, MitoSciences), and αtubulin (Santa Cruz).

### Statistical Tests

Statistical significance was determined by ANOVA and Newman-Keuls pairwise comparisons for multivariate experiments and t-test for experiments with 2 groups.

## Results

### Necroptosis does not occur in *Bax/Bak1* DKO MEFs

**Fig 1:**
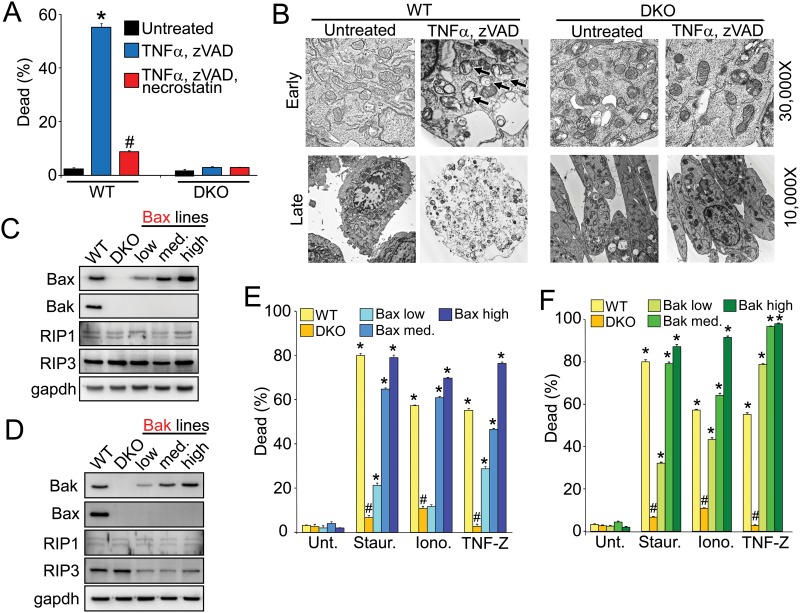
Bax and Bak are required for necroptosis. A, WT and *Bax/Bak1* DKO MEFs treated with TNFα and zVAD-FMK with and without necrostatin. Dead cells (cells with permeabilized membranes) were labeled with the Muse Count & Viability assay and were quantitated using a Muse Cell Analyzer. B, Transmission electron microscopy of WT and DKO MEFs treated with and without TNFα and zVAD-FMK for 2 hours (Early) and 12 hours (Late). Arrows show unhealthy mitochondria. Magnification used is shown. C, D, Western blots for Bax, Bak, RIP1, RIP3, and gapdh (control) from extracts of WT, DKO, and clonal DKO MEFs stably expressing Bax (C) or Bak (D). E, F, Cell death in WT, DKO, and clonal DKO MEFs stably expressing Bax (E) or Bak (F) treated with apoptotic inducer staurosporine (Staur.), necrotic inducer ionomycin (Iono.) or necroptotic inducer TNFα and zVAD-FMK (TNF-Z). All assays were performed in duplicate and averaged from three independent experiments. *p<0.01 vs. untreated; # p<0.01 vs. WT treatment.

We first evaluated the ability for *Bax/Bak1* double knock-out (DKO) mouse embryonic fibroblasts (MEFs) to die by necroptosis. Wildtype (WT) MEFs showed high levels of killing induced by the classical necroptotic inducers, TNFα in the presence of the pan caspase inhibitor zVAD-fmk, which was inhibited by the RIP1 blocking drug necrostatin-1 (Fig 1A). Similar to protection from apoptosis and MPTP-dependent necrosis, *Bax/Bak1* DKO MEFs were also highly resistant to necroptotic cell death (Fig 1A). Electron microscopy showed that WT MEFs treated with TNFα and zVAD-fmk displayed hallmarks of necrotic cell death; swollen mitochondria (arrows) at early time points followed later by plasma membrane rupture, with the absence of apoptotic morphologic features (Fig 1B). However, DKO MEFs displayed no signs of necrotic death or mitochondrial swelling or any other ultrastructural features of disorganization (Fig 1B).

To safeguard against an epiphenomenon associated with the clonally derived DKO MEFs we generated 6 additional clonal DKO lines with replacement of either Bax or Bak at low, medium and high levels of protein expression (Fig 1C and 1D). Importantly, all DKO lines expressed RIP1 and RIP3 at comparable levels to the DKO parent cell line, although RIP3 was slightly downregulated in cell lines with Bak reconstitution (Fig 1C and 1D). With respect to cell death, restoration of Bax or Bak in the DKO parent cell line produced a dosage-dependent rescue of apoptosis due to staurosporine, necrosis due to ionomycin or necroptosis due to TNFα and zVAD-fmk (Fig 1E and 1F). This strategy negates any potential secondary issues associated with the DKO MEF parent line and shows that Bax/Bak are required for necroptotic cell death.

### Necroptosis is dependent on the oligomerization Bax/Bak

**Fig 2:**
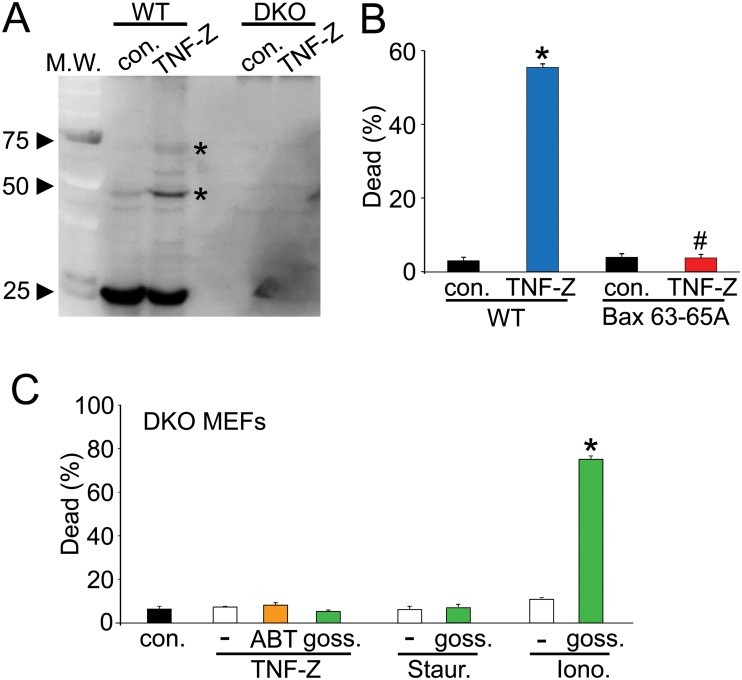
Bax/Bak oligomerization is required for necroptosis. A, Western blot for Bak from extracts of WT and *Bax/Bak1* DKO MEFs treated with and without TNFα and zVAD-FMK (TNF-Z) for 6 hours. In order to detect Bak oligomerization the extracts were processed under non-reducing conditions. Asterisks show Bak oligomers, and con. represents control conditions without stimulation. B, Quantitation of dead cells in WT and oligomerization dead Bax (Bax 63–65A) stably expressing DKO MEFs treated with TNFα and zVAD-FMK with and without necrostatin. C, Quantitation of dead cells in DKO MEFs pretreated with and without ABT-737 (ABT, in orange) or gossypol (goss., in green) and then treated with the necroptotic inducer TNFα and zVAD-FMK (TNF-Z), the apoptotic inducer staurosporine (Staur.), or the necrotic inducer ionomycin (Iono.). All assays were performed in duplicate and averaged from three independent experiments. *p<0.01 vs control; #<p<0.01 vs WT treatment.

Induction of necroptosis with TNFα and zVAD-fmk induced Bak oligomers in WT MEFs, suggesting a partial apoptotic-like mechanism (Fig 2A). In addition, DKO MEFs that stably expressed the oligomerization-dead mutant form of Bax (Bax 63–65A) were resistant to TNFα and zVAD-fmk induced necroptosis, again showing a more apoptotic mode of action for how Bak/Bak might induce cell death, but distinct from regulated necrosis where Bax monomers are sufficient for MPTP-dependent cell death (Fig 2B). As further proof of this mechanistic distinction we examined the effects of the BH-3 mimetics ABT-737 and gossypol on TNFα and zVAD-fmk induced cell death in DKO MEFs. As we previously showed [6], gossypol, but not ABT-737, restored ionomycin-induced necrosis but not staurosporine-induced apoptosis in the DKO MEFs (Fig 2C). Gossypol had no effect on DKO MEFs treated with TNFα and zVAD-fmk, similar to staurosporine (Fig 2C). These results indicate that necroptosis requires some level of Bax and Bak oligomerization to produce cell death, and that simply increasing the permeability characteristics of the outer membrane with gossypol is not sufficient to restore cell death induced by TNFα and zVAD-fmk. Finally, the fact that ABT-737 had no effect on necroptosis in DKO MEFs rules out compensation by any sort of anti-apoptotic effect of Bcl-2 family members in the absence of Bax and Bak at the outer membrane. Thus, necroptosis requires Bax/Bak through a MOMP mechanism of action.

### Necroptosis does not occur efficiently in *Ppif* null MEFs

**Fig 3:**
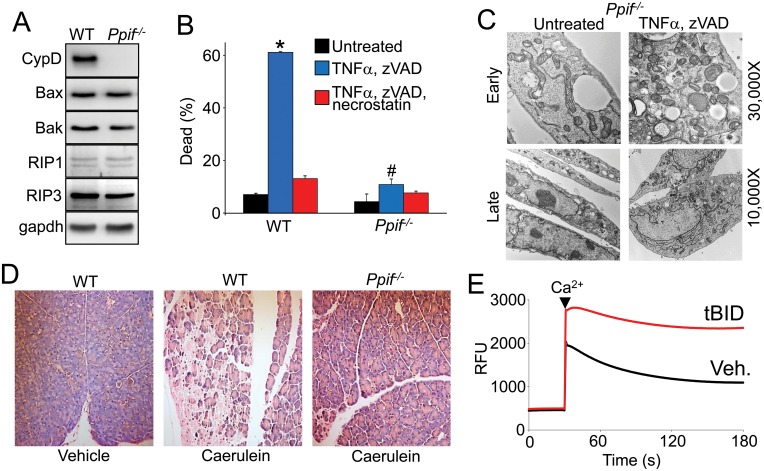
MPTP opening is required for necroptosis. A, Western blots for CypD, Bax, Bak, RIP1, RIP3, and gapdh (control) from extracts of WT and *Ppif* ^*-/-*^ MEFs. B, Quantitation of dead cells in WT and *Ppif* ^*-/-*^ MEFs treated with TNFα and zVAD-FMK with and without necrostatin. All assays were performed in duplicate and averaged from three independent experiments. *p<0.01 vs untreated; # p<0.01vs WT treated. C, Transmission electron microscopy in *Ppif* ^*-/-*^ MEFs treated with and without TNFα and zVAD-FMK for 2 hours (Early) and 12 hours (Late) at 2 different magnifications. D, Images of pancreas stained with H&E from WT and *Ppif* ^*-/-*^ mice treated with caerulein to induce necroptosis-mediated pancreatitis (200X magnification). E, Calcium uptake capacity assay with membrane impermeable calcium indicator dye Calcium Green-5N in purified mitochondria from WT liver pretreated with tBID (red) or vehicle (black). The calcium addition is labeled with an arrowhead. Fluorescence reduces as the mitochondria take up the calcium from the solution; three independent experiments were performed, although a representative trace is shown.

We previously showed that Bax and Bak can also influence necrotic cell death through the MPTP [6], hence here we employed *Ppif* ^*-/-*^ SV40 transformed MEFs and *Ppif* ^*-/-*^ mice to assess the involvement of the MPTP as a putative downstream necroptotic effector pathway. We first verified that the necroptotic regulators RIP1 and RIP3 were equally expressed in *Ppif* ^*-/-*^ MEFs, as were Bax and Bak (Fig 3A). *Ppif* ^*-/-*^ MEFs are partially resistant to necrosis induced by ionomycin or H_2_O_2_, but not against apoptosis induced by staurosporine [3, 4, 6]. *Ppif* ^*-/-*^ MEFs were also partially resistant to necroptosis induced by TNFα and zVAD-fmk compared with WT MEFs (Fig 3B). Furthermore, like the DKO MEFs, *Ppif* ^*-/-*^ MEFs lacked the ultrastructural signs of necrotic cell death observed in WT MEFs after TNFα and zVAD (Figs 3C, versus 1B). To examine if this result could be translated to an in vivo setting we treated WT and *Ppif* ^*-/-*^ mice with caerulein to cause necroptotic cell death in the pancreas. Caerulein treatment generates a model of necroptosis, supported by the observation that both *Ripk3* and *Mlkl* null mice are resistant to cell death induced by this agent [13] [15]. Remarkably, *Ppif* ^*-/-*^ mice were resistant to caerulein-induced cell death in the pancreas compared to WT mice (Fig 3D). To more carefully address the potential mechanism here we treated isolated liver mitochondria with and without tBID to induce Bax/Bak oligomerization/MOMP, and then subjected them to a calcium stress. Calcium uptake in the mitochondria treated with tBID was inhibited suggestive of greater MPTP opening when compared to mitochondria without tBID given the same amount of calcium (Fig 3E). These results suggest that the Bax/Bak oligomerization that occurs during necroptosis can sensitize a cell to die by MPTP-dependent cell death simply by increasing the permeability of the outer mitochondrial membrane.

### Upstream necroptotic regulators signal through mitochondrial death modifiers

**Fig 4:**
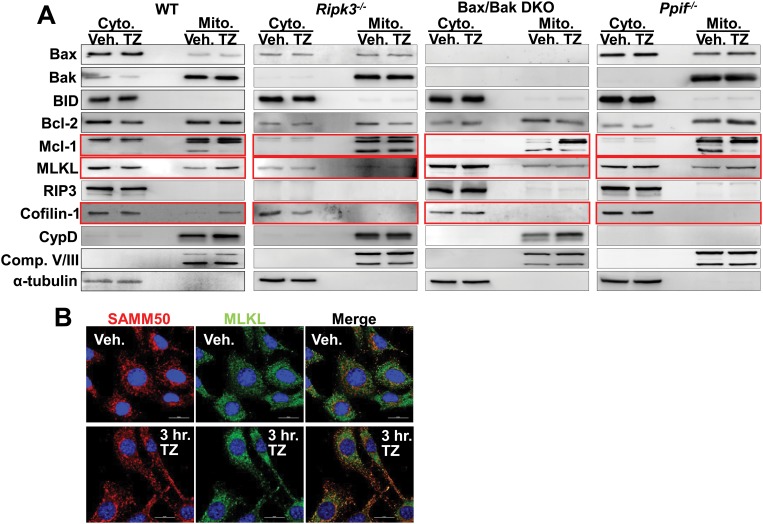
Upstream necroptotic regulators signal to the mitochondrion through Mcl-1. A, Western blots for Bax, Bak, BID, Bcl-2, Mcl-1, MLKL, RIP3, cofilin-1, CypD, Complex V and III (mitochondrial loading control), and αtubulin (cytoplasmic loading control) from cytoplasmic and mitochondrial extracts from WT, *Ripk3* null, *Bax/Bak1* DKO, and *Ppif* null MEFs treated with TNFα and zVAD-FMK (TZ) or vehicle (Veh.) for 6 hours. B, Immunocytochemistry for MLKL (green) and mitochondrial protein SAMM50 (red) on WT MEFs treated with TNFα and zVAD-FMK (TZ) or vehicle (Veh.) for 3 hours. Nuclei are stained with Dapi (blue).

It is known that RIP1, RIP3 and MLKL are the integral regulators of necroptotic cell death but it is unclear how these effectors might connect downstream to the MOMP or the MPTP. To address this issue we isolated mitochondria from WT, *Ripk3* null, *Bax/Bak1* DKO and *Ppif* null MEFs treated with and without TNFα and zVAD-fmk and probed for known regulators of necroptosis and the MOMP and MPTP (Fig 4A). We found that upon treatment of TNFα and zVAD-fmk the expression of MLKL increases in the mitochondrial fraction (Fig 4A). To confirm this result we performed immunocytochemistry for MLKL and SAMM50 (mitochondrial marker), which showed greater translocation to the mitochondria in WT MEFs treated with TNFα and zVAD-fmk compared with vehicle stimulation (Fig 4B). However, necroptosis-induced translocation of MLKL to the mitochondria was not observed in the *Ripk3* ^*-/-*^ MEFs suggesting that this event required RIP3 activity (Fig 4A). Less MLKL translocation to the mitochondria was also observed in *Bax/Bak1* DKO MEFs with TNFα and zVAD-fmk (Fig 4A).

Upon examination of an array of Bcl-2 family members we found only 1 to be changed during necroptosis. The mitochondrial matrix isoform of Mcl-1, which is normally thought to be protective [23], was mostly lost upon necroptosis but this loss was inhibited by deletion of *Ripk3* suggesting that necroptosis can have a direct effect on mitochondrial death regulatory proteins (Fig 4A). However, deletion of *Bax/Bak1* or *Ppif* did not antagonize the loss of Mcl-1 from the mitochondrial matrix (Fig 4A). Another protein that translocated to the mitochondria in WT MEFs and not in *Ripk3* ^*-/-*^ MEFs upon TNFα and zVAD-fmk treatment was cofilin-1 (Fig 4A). Cofilin-1 is an actin depolymerase, but in times of stress it translocates to the mitochondria where it can lead to mitochondrial dysfunction [20–22]. For example, isolated mitochondria treated with recombinant oxidized cofilin-1 induces MPTP dependent mitochondrial swelling [22]. Here we observed that cofilin-1 translocation did not occur in the *Bak/Bak1* DKO or *Ppif* null MEFs following treatment of TNFα and zVAD-fmk, suggesting cofilin-1 translocation is downstream of mitochondrial dysfunction and that it could also participate in necroptosis.

## Discussion

**Fig 5:**
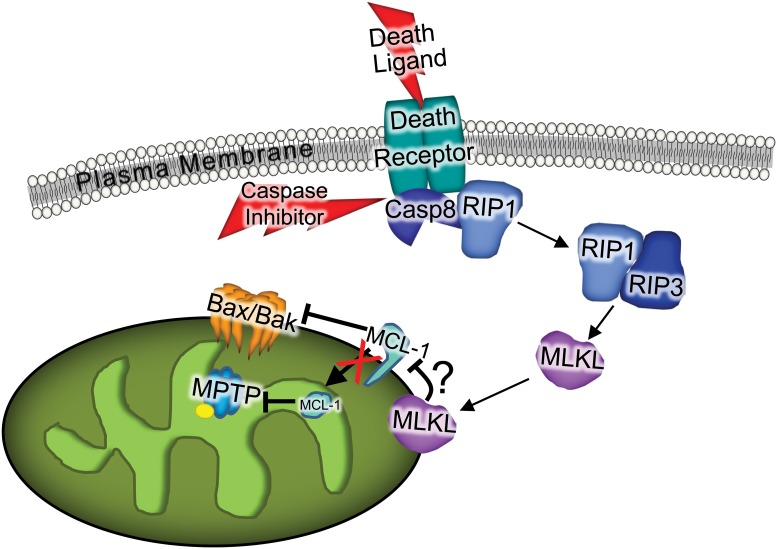
The necroptotic pathway and mitochondrial involvement. Schematic representation of the necroptotic pathway showing that upon treatment of TNFα and zVAD-FMK, upstream effectors RIP1 and RIP3 lead to MLKL translocation to the mitochondria where matrix Mcl-1 is depleted and the MOMP and MPTP are engaged, thus leading to mitochondrial dysfunction that is necessary for effective cell death through necroptosis.

Bax/Bak lead to two permeability states of the outer mitochondrial membrane, a low permeability state produced by inactive monomeric Bax/Bak and a high permeability state created by active oligomeric Bax/Bak that is responsible for MOMP and the release of cytochrome *c*. In addition, the lower permeability state induced by Bax/Bak monomers is sufficient to allow mitochondrial swelling and rupture with ionomycin treatment, leading to a type of regulated necrotic cell death through the MPTP. Here, we show that necroptosis involves the mitochondria in mediating cell killing, but through a primary mechanism involving Bax/Bak oligomerization in the outer mitochondrial membrane that in turn increases the sensitivity of MPTP opening to any given calcium or ROS stimuli, which then produces the characteristic necrotic signature of the cells as visualized by electron microscopy. Therefore, necroptosis is a hybrid of apoptosis and necrosis as it utilizes both mitochondrial MOMP and MPTP in mediating how the cell actually dies (Fig 5). Furthermore, we show that the upstream regulators of the necroptotic pathway impact the protein content of the matrix isoform of Mcl-1, which could participate as a triggering event of mitochondrial dysfunction (Fig 5).

Here we show that mitochondrial dysfunction is a necessary downstream mechanism whereby necroptosis produces cell death, as *Ppif* or *Bax/Bak1* null MEFs maintain mitochondrial integrity and are protected from cell death with TNFα and zVAD-fmk treatment. These results are consistent with a recent manuscript showing that ablation of the mitochondria through treatment of CCCP and Parkin overexpression has no effect on necroptosis while severely blunting apoptosis, as apoptosis requires cytochrome-c release and significant ATP to proceed [18]. Hence, maintenance of mitochondrial function in the face of necrotic stimuli serves to protect the cell from death, as in either *Ppif* or *Bax/Bak1* DKO null MEFs. These results explain some of the ongoing controversy in the necroptosis field in regards to mitochondrial involvement [16]. In the case of necroptosis, death receptor activation in the presence of caspase inhibition leads to necrosis [27] that others have subsequently shown utilizes a RIP-dependent mechanism of cell killing [12, 13]. However, it remains unclear and controversial how RIP pathway activation directly leads to cell killing. Here we suggestively linked the necroptotic RIP effectors to these 2 processes by showing MLKL and cofilin-1 translocation to mitochondria as well as showing depleted matrix Mcl-1 following necroptosis induction, effects that are inhibited in *Ripk3* null MEFs.

One inconsistency with our proposed model is based on the observation that caspase 8 deficient mice, which die during embryogenesis due to aberrant necroptotic cell death, are not rescued by simultaneous deletion of the *Ppif* gene [18]. In contrast, embryonic lethality associated with caspase 8 deficiency is fully rescued by deletion of *Ripk3* in mice [28]. Our data show that MOMP occurs during necroptosis and that *Ppif* null cells are resistant to death induced by TNFα and zVAD-fmk. During this treatment all caspase activity is blocked with zVAD-fmk while in caspase 8 null mice other caspases remain functional. Since the MOMP presumably takes place downstream of RIP activation, death receptor stimulation in the absence of caspase 8 and CypD protein may result in apoptotic cell death *in vivo* as mitochondrial function is preserved and all other caspases are unaffected. Another reason why the *Ppif* caspase 8 double null mice may not be viable is that the MPTP may still be engaging in the absence of CypD, as loss of this protein only desensitizes opening and extreme stimuli can still cause necrosis [29].

Many questions remain as to how TNFα and zVAD-fmk treatment leads to mitochondrial dysfunction following RIP activation, although it is clear that mitochondrial preservation is protective during a necroptotic stress. The role of MLKL at the mitochondria needs to be further elucidated since this protein is required for necroptosis and it translocates to the mitochondria following a necroptotic stress. Furthermore, cofilin-1 translocation and matrix Mcl-1 depletion are two intriguing mechanisms that may be initiating events leading to mitochondrial dysfunction during necroptosis, as both events are known to cause mitochondrial dysfunction. Overall our results support the hypothesis that mitochondrial dysfunction is a critical step required for cell death due to upstream necroptotic signaling. Thus, necroptosis is a unique form of cell death that incorporates downstream mechanistic effectors of apoptotic (MOMP) and necrotic (MPTP) pathways.
